# Characteristics and distribution of *Listeria* spp., including *Listeria* species newly described since 2009

**DOI:** 10.1007/s00253-016-7552-2

**Published:** 2016-04-29

**Authors:** Renato H. Orsi, Martin Wiedmann

**Affiliations:** Department of Food Science, Cornell University, Ithaca, NY 14853 USA

**Keywords:** *Listeria*, *Listeria* sensu strictu, *Listeria* sensu lato, New species, New genus

## Abstract

The genus *Listeria* is currently comprised of 17 species, including 9 *Listeria* species newly described since 2009. Genomic and phenotypic data clearly define a distinct group of six species (*Listeria* sensu strictu) that share common phenotypic characteristics (e.g., ability to grow at low temperature, flagellar motility); this group includes the pathogen *Listeria monocytogenes*. The other 11 species (*Listeria* sensu lato) represent three distinct monophyletic groups, which may warrant recognition as separate genera. These three proposed genera do not contain pathogens, are non-motile (except for *Listeria grayi*), are able to reduce nitrate (except for *Listeria floridensis*), and are negative for the Voges-Proskauer test (except for *L. grayi*). Unlike all other *Listeria* species, species in the proposed new genus *Mesolisteria* are not able to grow below 7 °C. While most new *Listeria* species have only been identified in a few countries, the availability of molecular tools for rapid characterization of putative *Listeria* isolates will likely lead to future identification of isolates representing these new species from different sources. Identification of *Listeria* sensu lato isolates has not only allowed for a better understanding of the evolution of *Listeria* and virulence characteristics in *Listeria* but also has practical implications as detection of *Listeria* species is often used by the food industry as a marker to detect conditions that allow for presence, growth, and persistence of *L. monocytogenes*. This review will provide a comprehensive critical summary of our current understanding of the characteristics and distribution of the new *Listeria* species with a focus on *Listeria* sensu lato.

## Introduction

The genus *Listeria* currently includes 17 recognized species (*Listeria monocytogenes*, *Listeria seeligeri*, *Listeria ivanovii*, *Listeria welshimeri*, *Listeria marthii*, *Listeria innocua*, *Listeria grayi*, *Listeria fleischmannii*, *Listeria floridensis*, *Listeria aquatica*, *Listeria newyorkensis*, *Listeria cornellensis*, *Listeria rocourtiae*, *Listeria weihenstephanensis*, *Listeria grandensis*, *Listeria riparia*, and *Listeria booriae*) of small rod-shaped gram-positive bacteria. Only two of these species, *L. monocytogenes* and *L. ivanovii*, are considered pathogens. *L. monocytogenes* is an important human foodborne pathogen and the third leading cause of foodborne deaths due to microbial causes in the USA (Scallan et al. 2011). Human disease cases and outbreaks caused by this organism have a considerable economic impact for society and the food industry (Ivanek et al. 2004). In addition, the food industry as well as regulatory agencies around the world perform a large number of tests, of food and environmental samples, for *L. monocytogenes* and *Listeria* spp.; hence, sales of test kits for these organisms are an important revenue for a number of companies. Importantly, detection of *Listeria* species is often used by the food industry as a marker to detect conditions that allow for presence, growth, and persistence of *L. monocytogenes*. Hence, identification of new *Listeria* species and changes in the taxonomy of *Listeria* can have considerable impacts on food industry and test kit manufacturers.

Eleven *Listeria* species (*L. marthii*, *L. rocourtiae*, *L. weihenstephanensis*, *L. grandensis*, *L. riparia*, *L. booriae*, *L. fleischmannii*, *L. floridensis*, *L. aquatica*, *L. newyorkensis*, and *L. cornellensis*) have been described since 2009 (Bertsch et al. 2013; den Bakker et al. 2014; Graves et al. 2010; Lang Halter et al. 2013; Leclercq et al. 2010; Weller et al. 2015). Before this, the last description of a new *Listeria* species occurred in 1984 (*L. ivanovii*) (Seeliger et al. 1984). Importantly, all these newly recognized species have been validly described through publications in the *International Journal of Systematic and Evolutionary Microbiology*, and their species classification is supported by recognized taxonomic criteria (ANib and/or DNA-DNA hybridization data). Similar to a previous report (Chiara et al. 2015), we will, in this review, categorize the species in the genus *Listeria* into two groups including (i) *Listeria* sensu strictu, which includes *L. monocytogenes*, *L. seeligeri*, *L. marthii*, *L. ivanovii*, *L. welshimeri*, and *L. innocua* (Chiara et al. 2015), and (ii) *Listeria* sensu lato group, which includes the other 11 *Listeria* species (*L. grayi* and 10 *Listeria* species newly described since 2009). The separation into these two groups is based on the relatedness of species to *L. monocytogenes*, which is both the first named *Listeria* species classified and the most important species in terms of public health and economic impact. This review will provide a summary of the current knowledge on the phenotypic and genetic characteristics of all members of the genus *Listeria* with a focus on *Listeria* sensu lato and the *Listeria* species that have been newly described since 2009. Insights into the recently recognized expanded diversity of the current genus *Listeria* will both improve our ability to understand the evolution of virulence and virulence-associated characteristics in this important gram-positive group and will help with an improved ability to develop and implement testing procedures for both *L. monocytogenes* and *Listeria* species.

## *Listeria* sensu strictu

**Fig. 1:**
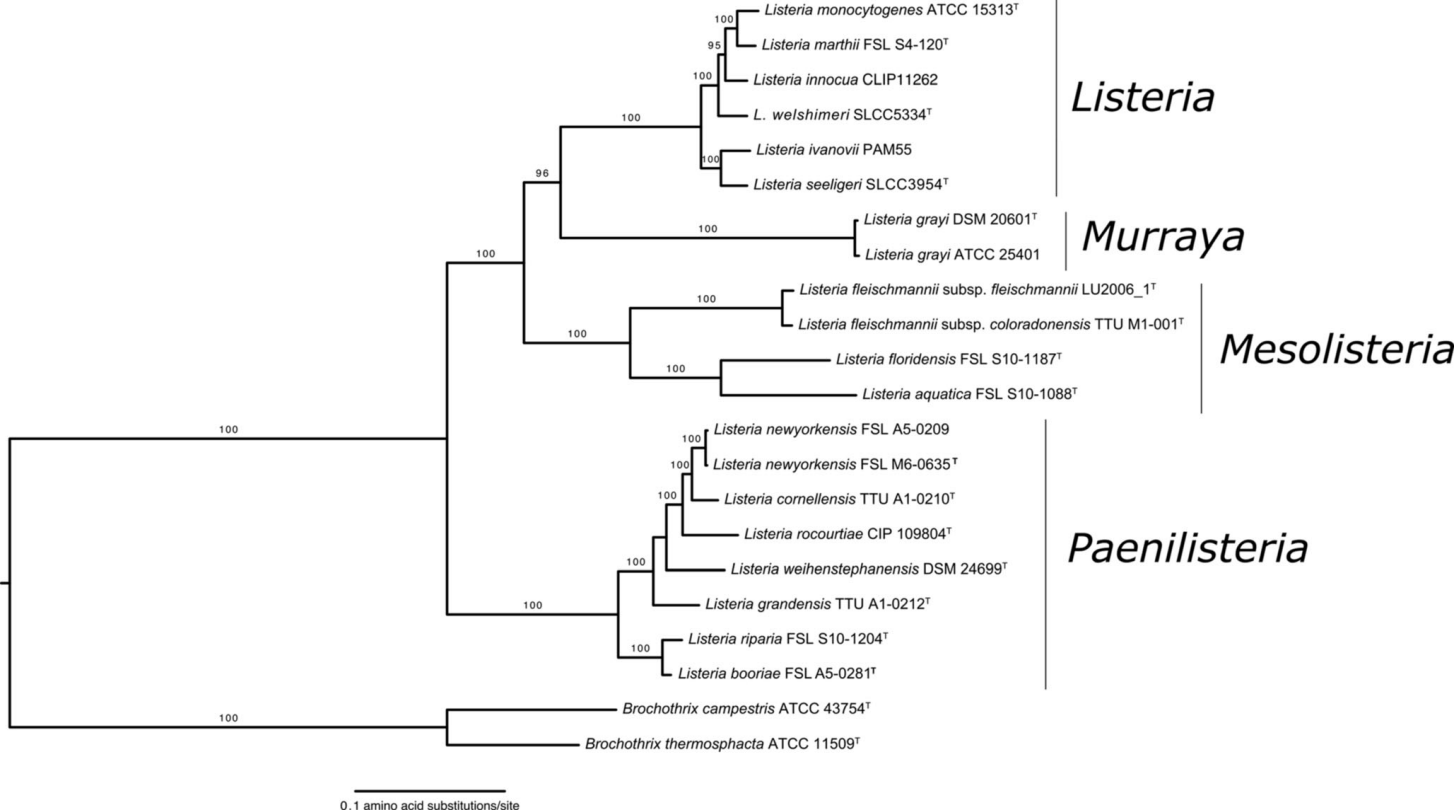
Phylogenetic tree modified from Weller et al. (2015). Maximum likelihood phylogeny based on concatenated amino acid sequences of 325 single copy genes present in all *Listeria* species. *Values on branches* represent bootstrap values (>70 %) based on 250 bootstrap replicates. Proposed new genera names are shown close to monophyletic groups. *Bar*, 0.1 amino acid substitutions per site

*Listeria* sensu strictu includes *L. monocytogenes*, *L. seeligeri*, *L. ivanovii*, *L. welshimeri*, and *L. innocua*, which all have been described before 1985 as well as *L. marthii*, which was first described in 2010. *Listeria* sensu strictu species form a tight monophyletic group within the genus *Listeria* (Fig. 1). Except for *L. monocytogenes* and *L. innocua*, all species in this group have been named after researchers that have played important roles in the study of *Listeria*.

## Distribution

Most species within *Listeria* sensu strictu are well documented to be widely distributed and commonly found in different environments. *L. monocytogenes* has been isolated throughout the world, including in North America (Chapin et al. 2014; Sauders et al. 2012; Stea et al. 2015), South America (Hofer et al. 2000; Montero et al. 2015; Ruiz-Bolivar et al. 2011; Vallim et al. 2015), Europe (Gnat et al. 2015; Linke et al. 2014), Asia (Huang et al. 2015; Sugiri et al. 2014; Tango et al. 2014), Africa (Ahmed et al. 2013; Hmaied et al. 2014; Ndahi et al. 2014), and Oceania (McAuley et al. 2014). Human listeriosis cases have been reported throughout the world, including Africa, Oceania, South America, North America, Europe, and Asia (Ariza-Miguel et al. 2015; Barbosa et al. 2015; Hmaied et al. 2014; Huang et al. 2015; Lomonaco et al. 2015; Najjar et al. 2015; Negi et al. 2015; Ramdani-Bouguessa and Rahal 2000; Scallan et al. 2015a; Scallan et al. 2015b). Similarly, animal listeriosis cases caused by *L. monocytogenes* have also been reported throughout the world, including Africa (Akpavie and Ikheloa 1992; Meredith and Schneider 1984; Schroeder and van Rensburg 1993), South America (Headley et al. 2013; Headley et al. 2014), North America (Wiedmann et al. 1997; Wiedmann et al. 1994; Wiedmann et al. 1999; Woo-Sam 1999), Asia (Gu et al. 2015; Malik et al. 2002), Oceania (Fairley and Colson 2013; Fairley et al. 2012), and Europe (Rocha et al. 2013; Vela et al. 2001; Wagner et al. 2005). Animal listeriosis cases due to *L. ivanovii* has been recorded in different continents, including Oceania (McAuley et al. 2014; Sergeant et al. 1991), North America (Alexander et al. 1992), Europe (Gill et al. 1997; Kimpe et al. 2004), South America (Hofer et al. 2000), and Asia (Chand and Sadana 1999). We were not able to identify any reports of animal listeriosis caused by *L. ivanovii* in Africa. While *L. ivanovii* has rarely been linked to human listeriosis, *L. ivanovii* isolation from humans with listeriosis symptoms has been reported in different continents, including Europe (Cummins et al. 1994; Guillet et al. 2010; Lessing et al. 1994) and Asia (Snapir et al. 2006). We were unable to identify any reports of human listeriosis caused by *L. ivanovii* in Africa, Oceania, South America, and North America.

*L. innocua*, *L. seeligeri*, and *L. welshimeri* have also been identified throughout the world in most studies that identified reasonable large sets of *Listeria* isolates that were not *L. monocytogenes* to the species level (Chapin et al. 2014; Fox et al. 2015; Hofer et al. 2000; Linke et al. 2014; Sauders et al. 2012; Stea et al. 2015). A number of these studies have often failed to isolate *L. ivanovii* from environmental samples (Chapin et al. 2014; Fox et al. 2015; Sauders et al. 2012). For example, Sauders et al. (2012) reported that 23.4 and 22.3 % of samples obtained from natural and urban environments in New York State (USA), respectively, were positive for *Listeria*; the 442 *Listeria* isolates characterized in this study represented *L. seeligeri* (234 isolates), *L. monocytogenes* (80 isolates), *L. welshimeri* (74 isolates), *L. innocua* (50 isolates), and *L. marthii* (4 isolates). In a study in Austria (Linke et al. 2014), 149 out of 467 soil samples (30 %) were positive for *Listeria* spp.; species identified included *L. monocytogenes* (6 % of all samples), *L. seeligeri* (15 % of samples), *L. innocua* (6 % of samples), *L. ivanovii* (3 % of samples), *L. welshimeri* (2 % of samples), and unidentified *Listeria* spp. (2 % of samples). *L. seeligeri* thus has been the most commonly isolated *Listeria* species in two largest studies on *Listeria* diversity in natural environments. In a study in Canada (Stea et al. 2015), *Listeria* spp. were isolated from 53.8 % of the 329 water samples with a detection rate of 72.1 % in rural watersheds compared to 35.4 % for urban watersheds. *L. monocytogenes* was found in 30.3 and 34.5 % of the positive rural and urban watershed samples, respectively, and *L. ivanovii* was found in 8.4 and 6.9 % of these samples. Other *Listeria* spp. were not individually identified in this study but grouped into two large groups. The *L. innocua* group, including *L. innocua*, *L. seeligeri*, *L. marthii*, and *L. grayi*, was isolated from 56.3 and 34.5 % of the positive rural and urban watershed samples, respectively. The *L. welshimeri* group, which included *L. welshimeri* and all other *Listeria* spp., was isolated from 37 and 43.1 % of the positive rural and urban watershed samples, respectively (Stea et al. 2015). Despite the less frequent isolation of *L. ivanovii*, this species has been isolated from farm environments (McAuley et al. 2014). While these data indicate that *L. ivanovii* is one of the least commonly isolated *Listeria* sensu strictu species, this could also be due to reduced recovery of this species in the enrichment media and isolation protocols that have typically been optimized for recovery of *L. monocytogenes*.

Conversely, the one *Listeria* sensu strictu species that may not be globally distributed is *L. marthii*. Thus far, this species has only been isolated from very specific natural areas in a small part of New York State (USA), Connecticut Hill and Finger Lakes National Forest, which are natural areas in close proximity to each other (Chapin et al. 2014; Sauders et al. 2012). In these same studies, *L. marthii* was not found in other natural areas and urban areas (Chapin et al. 2014; Sauders et al. 2012). A PubMed search as of November 11, 2015 also was not able to locate other studies that have reported identification of *L. marthii*. This could at least be partially due to the fact that *L. marthii* cannot be easily distinguished from the closely related *L. innocua* and thus could have been misclassified in other studies.

In general, there is no clear indication that different *Listeria* sensu strictu species are preferably found in certain environments, even though individual studies have identified statistical associations between different environments and isolation of specific *Listeria* species. For example, a study in the state of New York (USA) reported that *L. seeligeri* and *L. welshimeri* were significantly associated with natural environments (*P* < 0.0001), while *L. innocua* and *L. monocytogenes* were significantly associated with urban environments (*P* < 0.0001) (Sauders et al. 2012). A study carried out in Austria (Linke et al. 2014) found a significant association between the presence of *Listeria* spp. in soil samples and abiotic conditions such as moisture, pH, and soil type. The authors specifically found that *Listeria* spp. were more frequently isolated from soil samples with low moisture content, neutral pH, and soil types consisting of a mixture of sand and humus. The same authors also observed a seasonal effect on the prevalence of *Listeria* spp. in soil with the lowest isolation rates observed in July (winter).

## Phenotypic characteristics

*Listeria* sensu strictu species share many phenotypic characteristics; this makes isolates from this group easily recognizable as being members of the genus *Listeria* (Table 1). Key phenotypic characteristics shared by all *Listeria* sensu strictu species include (i) ability to grow at temperatures as low as 4 °C, (ii) motility (at least at 30 °C), (iii) positive catalase reaction, (iv) inability to reduce nitrate to nitrite, and (v) positive reaction in the Voges-Proskauer test, indicating ability to produce acetoin from the fermentation of glucose through the butanediol pathway. Moreover, all sensu strictu species are capable of fermenting D-arabitol, α-methyl D-glucoside, cellobiose, D-fructose, D-mannose, N-acetylglucosamine, maltose, and lactose, while none of the species can ferment inositol, L-arabinose, and D-mannitol (Bertsch et al. 2013; Bille et al. 1992; den Bakker et al. 2014; McLauchlin and Rees 2009; Weller et al. 2015).

**Table 1:** Phenotypic characteristics of *Listeria* species (modified from Weller et al. 2015)

*Listeria* species	Voges-Proskauer	Methyl red	Nitrate reduction	Motility	Growth at 4 °C	Hemolysis	PI-PLC	Arylamidase	α-Mannosidase	Fermentation of	D-Arabitol	D-Xylose	L-Rhamnose	α-Methyl-D-glucoside	D-Ribose	Glucose-1-phosphate	D-Tagatose	D-Mannitol	Sucrose	Turanose	Glycerol	D-Galactose	L-Arabinose	L-Sorbose	Inositol	Methyl-α-D-mannose	Maltose	Lactose	Melibiose	Inulin	D-Melezitose	D-Lyxose	D-Glucose
*Listeria* sensu stricto																																	
*L. monocytogenes*	+	+	−	+	+	+	+	−	+		+	−	+	+	−	−	−	−	+	−	V	V	−	V!	−	−	+	+	V!	V!	V	V	V!
*L. marthii*	+	+	−	+	+	−	−	−	+		+	−	−	+	−	−	−	−	−	+	−	−	−	V!	−	−	+	+	V	−	−	−	V!
*L. innocua*	+	+	−	+	+	−	−	+	+		+	−	V	+	−	−	−	−	+	V	+	−	−	V!	−	−	+	+	V	V!	V	V	V!
*L. welshimeri*	+	+	−	+	+	−	−	V	+		+	+	V	+	−	−	+	−	+	−	+	−	−	−	−	ND	+	+	−	−	V	V	+
*L. ivanovii*	+	+	−	+	+	+	+	V	−		+	+	−	+	+	V	−	−	+	−	+	V	−	V!	−	−	+	+	−	−	V	−	V!
*L. seeligeri*	+	+	−	+	+	+	−	+	−		+	+	−	+	−	−	−	−	+	−	+	−	−	−	−	ND	+	+	−	−	V	−	+
*Listeria* sensu lato, possible new genus *Murraya*																																	
*L. grayi*	+	+	V	+	+	−	−	+	V		+	−	−	+	+	−	−	+	−	−	V	+	−	V!	−	+	+	+	−	−	−	V	+
*Listeria* sensu lato, possible new genus *Mesolisteria*																																	
*L. fleischmannii*	−	+	+	−	−	−	−	−	−		+	+	+	+	+	−	−	V	V	V	+	−	−	V	V	V	+	+	V	−	V	−	+
*L. floridensis*	−	+	−	−	−	−	−	−	−		−	+	+	+	−	−	−	−	−	−	−	+	+	−	−	−	+	+	−	−	−	+	+
*L. aquatica*	V	+	+	−	−	−	−	−	+		−	+	+	−	+	−	+	−	−	−	V	−	+	−	V	−	−	−	−	−	−	V	+
*Listeria* sensu lato, possible new genus *Paenilisteria*																																	
*L. newyorkensis*	−	+	+	−	+	−	−	−	−		−	+	V	+	+	−	−	+	−	−	+	+	+	−	−	−	+	+	−	−	−	−	+
*L. cornellensis*	−	V	+	−	+	−	−	−	−		−	+	−	+	+	−	−	−	−	−	V	−	V	−	−	−	+	(+)	−	−	−	−	+
*L. rocourtiae*	−	V	+	−	+	−	−	−	+		−	+	+	+	+	−	−	+	−	−	+	+	−	−	−	−	+	+	+	−	−	−	+
*L. weihenstephanensis*	−	V	+	−	+	−	−	−	−		+	+	+	+	−	−	−	+	−	−	+	−	−	−	−	−	+	V!	−	−	−	−	+
*L. grandensis*	−	+	+	−	+	−	−	−	−		V	+	−	+	+	−	−	−	−	−	−	−	−	−	−	−	+	−	−	−	−	−	+
*L. riparia*	−	V	+	−	+	−	−	−	+		−	+	+	+	V	−	−	V	−	−	V	+	+	−	V	−	+	+	V	−	−	−	+
*L. booriae*	−	+	+	−	+	−	−	−	+		+	+	+	+	V	−	−	+	−	−	+	+	+	−	−	−	+	+	+	−	−	−	+

*PI-PLC* phosphoinositide phospholipase C, *+* positive, − negative, *V* variable between replicates and/or strains, *V!* variable between studies, *ND* not determined or not recorded, (+) weekly positive

Only the two species considered pathogenic, *L. monocytogenes* and *L. ivanovii*, as well as a few *L. innocua* strains (Johnson et al. 2004; McLauchlin and Rees 2009) show phosphatidylinositol-specific phospholipase C (PI-PLC) activity, while *L. monocytogenes*, *L. ivanovii*, and *L. seeligeri* as well as some *L. innocua* strains show hemolytic capabilities (Johnson et al. 2004; McLauchlin and Rees 2009). *L. ivanovii* can be differentiated from *L. monocytogenes* by its unique ability, among sensu strictu species, to ferment D-ribose (Bertsch et al. 2013; Bille et al. 1992; den Bakker et al. 2014; McLauchlin and Rees 2009; Weller et al. 2015). *L. welshimeri* can be identified by its ability to ferment D-tagatose, while *L. seeligeri* is the only sensu strictu species capable of fermenting D-xylose but not capable of fermenting D-ribose or D-tagatose (Bertsch et al. 2013; Bille et al. 1992; den Bakker et al. 2014; McLauchlin and Rees 2009; Weller et al. 2015). *L. marthii* is the only sensu strictu species incapable of fermenting sucrose, a characteristic that can be used to differentiate strains from this species from other sensu strictu species (den Bakker et al. 2014). While *L. innocua* is typically identified by its inability to cause hemolysis and ferment D-xylose combined by its ability to ferment glycerol (Bertsch et al. 2013; Bille et al. 1992; den Bakker et al. 2014; McLauchlin and Rees 2009; Weller et al. 2015), hemolytic *L. innocua* strains are difficult to differentiate from *L. monocytogenes* and *L. seeligeri* based on standard biochemical tests (Johnson et al. 2004).

Importantly, phenotypic characteristics do not always allow for unambiguous classification of *Listeria* isolates. Phenotypic data alone, sometimes in combination with genetic data, have led to description of a number of “atypical” *Listeria* isolates both before and after 1985. For example, hemolytic *L. innocua* strains (as discussed above) could be considered atypical *Listeria*. Similarly, a number of atypical (including non-hemolytic) *L. monocytogenes* have been described (Burall et al. 2014; Hof 1984; Moreno et al. 2014; Pine et al. 1987). While one could argue that these non-hemolytic *L. monocytogenes* strains that were described before the taxonomic description of *L. marthii* may indeed have been *L. marthii*, a number of these strains were confirmed to truly be atypical *L. monocytogenes*. For example, the initial ATCC strain (ATCC 15313) which was found to be non-hemolytic (Jones and Seeliger 1983) appears to clearly represent *L. monocytogenes*. More recently, another non-hemolytic strain was confirmed as belonging to the species *L. monocytogenes* after whole genome sequencing (Burall et al. 2014). Overall, one cannot exclude though that at least some of the atypical *Listeria* strains that have been described in the past may represent species that either were not described at the times or have not been identified at all. Future characterization of atypical *Listeria* isolates by whole genome sequencing and phylogenetic analysis would help to resolve some of these identification challenges and may yield additional species that have not yet been described.

## Genomic characteristics

*Listeria* sensu strictu genomes share many characteristics. The G+C content varies from 34.6 to 41.6 %; genome sizes vary from 2.8 to 3.2 Mb, and *Listeria* genomes are highly syntenic (i.e., the order of the genes are highly conserved across different species) (den Bakker et al. 2010b). The *Listeria* sensu strictu pan-genome has been estimated as approximately 6500 genes, 17 % of which are involved in nucleobase, nucleotide, nucleoside, and nucleic acid metabolism, 14 % of which are involved in cellular macromolecular metabolism, and 10 % of which are involved in protein metabolic process (den Bakker et al. 2010b). The high number of internalin genes is another characteristic of *Listeria* sensu strictu genomes. *L. welshimeri* seems to have the lowest number of internalin genes among sensu strictu species with nine of these genes found in the only strain analyzed (den Bakker et al. 2010b). On the other hand, five different *L. monocytogenes* genomes were shown to have between 18 and 28 internalin genes encoded in their genomes (den Bakker et al. 2010b). Three *Listeria* pathogenicity islands (LiPI) have been identified in the genomes of *Listeria* sensu strictu strains. These LiPIs are further discussed below. Moreover, a 53-kb island that includes genes involved in the metabolism of ethanolamine, cobalamin, and propanediol has been identified in all *Listeria* sensu strictu species (Chiara et al. 2015). This island includes 82 genes involved in the cobalamin (vitamin B12) biosynthetic process and utilization of both ethanolamine and propane-2-diol, two carbon sources whose utilization is necessary for *L. monocytogenes* and *Salmonella* to cause disease in vertebrate hosts (Conner et al. 1998; Joseph et al. 2006; Klumpp and Fuchs 2007; Mellin et al. 2014). Absence of this island in all *Listeria* sensu lato species in conjunction with its homology and conserved arrangement of the genes in comparison to sequences from other bacteria led to speculations that the entire island has been transferred to *Listeria* sensu strictu ancestor through horizontal gene transfer from a *Salmonella*-like bacterium (Buchrieser et al. 2003) or from gram-positive bacteria (Chiara et al. 2015).

## Virulence

*L. monocytogenes* is a facultative intracellular pathogen that causes a rare, yet severe, human illness; typical symptoms include septicemia, abortions, and encephalitis (McLauchlin and Rees 2009). In addition, a few instances have been reported where *L. monocytogenes* infection of humans has led to gastrointestinal illnesses without systemic infections (Aureli et al. 2000; Dalton et al. 1997; Frye et al. 2002). Human listeriosis is virtually always caused by foodborne exposure to *L. monocytogenes* (Scallan et al. 2011). In addition to human disease, *L. monocytogenes* also has been reported to cause invasive disease in >40 animal species. *L. ivanovii* is also considered a pathogen but has been predominantly linked to animal disease with some studies suggesting that *L. ivanovii* predominantly infects sheep (Chand and Sadana 1999; Sergeant et al. 1991), but occasional isolation of *L. ivanovii* from bovines (Alexander et al. 1992; Gill et al. 1997) and humans with symptoms consistent with listeriosis suggests that this organism may also be able to cause disease in other animals, including rarely in humans (Cummins et al. 1994; Guillet et al. 2010; Lessing et al. 1994; Ramage et al. 1999; Snapir et al. 2006). In most human cases, *L. ivanovii* was isolated from the patient’s blood, with no clear evidence of the transmission route. However, at least in one case, the septicemic condition was preceded by a gastrointestinal illness suggesting that the strain was orally acquired (Guillet et al. 2010).

*L. innocua* was initially considered non-pathogenic (hence its name) and non-hemolytic. Recent work has however identified a number of hemolytic *L. innocua* isolates. Presumably, these isolates would have been (mis-)classified as *L. monocytogenes* before the use of molecular methods that allow for identification and phylogenetic characterization (e.g., MLST methods), which clusters hemolytic *L. innocua* strains with non-hemolytic *L. innocua*. Interestingly, some hemolytic *L. innocua* (e.g., FSL J1-023) have been shown to invade (human) Caco-2 cells at same levels as *L. monocytogenes* (den Bakker et al. 2010b), while another study has characterized the hemolytic *L. innocua* strain PRL/NW 15B95 as avirulent in a mouse model (Johnson et al. 2004). *L. innocua* has also been isolated at least once from a fatal human case (Perrin et al. 2003), further supporting that at least some *L. innocua* may be able to cause human disease. No information whether this strain was hemolytic or not was provided, though. *L. innocua* strains carrying a *L. monocytogenes* lineage I-specific pathogenicity island, LiPI-3, have also been found and shown to be hemolytic when the gene *llsA*, encoded within LiPI-3, was expressed from a constitutive highly expressed *Listeria* promoter (Clayton et al. 2014). One of these strains was isolated from a human patient with meningitis in the UK (Clayton et al. 2014). Whole genome sequencing has confirmed that hemolytic *L. innocua* strains contain a complete *Listeria* pathogenicity island 1 (also referred as the *prfA* cluster); specifically, the hemolytic isolate FSL J1-023 was found to contain a *Listeria* pathogenicity island 1 (LiPI-1) that shows a high level of homology with the *L. monocytogenes* pathogenicity island 1 (den Bakker et al. 2010a). This and genomically similar hemolytic *L. innocua* strains thus will test positive by PCR-based and other molecular methods that detect *L. monocytogenes* based on the presence of *hly* or other genes located in the pathogenicity island 1. Interestingly, this same hemolytic *L. innocua* isolate (FSL J1-023) did not contain the complete *inlAB* operon, which encodes two internalins (InlA and InlB) that are critical virulence factors, but only contained *inlA*. Other atypical *L. innocua* strains encoding LiPI-1 and *inlA* have been found in Brazil (Moreno et al. 2014) and in Korean-imported clams (Johnson et al. 2004), suggesting that these atypical *L. innocua* strains are widespread. The specific implications that the presence of *inlA* and the absence of *inlB* have on virulence in humans and other animals remain to be determined. While InlA is essential for invasion of human intestinal epithelial cells, InlB appears to contribute to invasion of human hepatic and placental cells (Lecuit 2007). While the data available to date suggest that at least some *L. innocua* may have the potential to cause invasive human disease, further data are thus needed to determine whether all or some hemolytic *L. innocua* are a human health hazard and what their virulence is relative to *L. monocytogenes*.

While isolates of the species *L. seeligeri* also typically are hemolytic, this species is generally considered non-pathogenic. Interestingly, non-hemolytic *L. seeligeri* strains have been reported (Volokhov et al. 2006), and one study suggested that non-hemolytic *L. seeligeri* represent a recent loss of the *Listeria* pathogenicity island 1 (*prfA* cluster) (den Bakker et al. 2010a). Some possible cases of human disease caused by *L. seeligeri* have been described, including a human meningitis case (Rocourt et al. 1986). Genomic studies suggest that hemolytic *L. seeligeri* contain a variant of the *Listeria* pathogenicity island 1 (*prfA* cluster), which is characterized by the presence of additional genes not found in *L. monocytogenes* and a partial duplication of the gene *plcB* (Schmid et al. 2005), which seems to have been split into two independent ORFs in some strains (den Bakker et al. 2010b). To date, the *inlAB* operon has not been found among *L. seeligeri* strains. A functional study (Stelling et al. 2010) on selected *L. seeligeri* virulence genes indicates that (i) regulation of *prfA* expression differs between *L. monocytogenes* and *L. seeligeri*, although *hly* transcription is temperature dependent in both species, and (ii) PrfA and Hly functions are largely, but not fully, conserved between *L. seeligeri* and *L. monocytogenes*. While virulence gene homologs and their expression thus appear to have adapted to distinct but possibly related functions in *L. monocytogenes* and *L. seeligeri* (Stelling et al. 2010), it remains to be determined whether *L. seeligeri* is pathogenic in specific, yet to be determined, host species. Despite rare isolation from humans with illness symptoms, there is no evidence that *L. seeligeri* should be considered a pathogen or presents a human health risk comparable to *L. monocytogenes*.

On the other hand, all *L. marthii* and *L. welshimeri* isolates characterized to date have been non-hemolytic. None of the (few) *L. marthii* and *L. welshimeri* genomes sequenced so far contain the *Listeria* pathogenicity island 1 (*prfA* cluster), further supporting that members of these two species are non-pathogenic.

## *Listeria* sensu lato

*Listeria* sensu lato is comprised of *L. grayi* (first described in 1966) as well as *L. fleischmannii*, *L. floridensis*, *L. aquatica*, *L. newyorkensis*, *L. cornellensis*, *L. rocourtiae*, *L. weihenstephanensis*, *L. grandensis*, *L. riparia*, and *L. booriae*. Phylogenetically, *L. grayi* is most closely related to *Listeria* sensu strictu species. *L. fleischmannii*, *L. floridensis*, and *L. aquatica* share a most recent common ancestor with *L. grayi* and sensu strictu species, while the other *Listeria* sensu lato species group together in the most basal cluster within the genus (Fig. 1; Weller et al. 2015). A list of *Listeria* sensu lato species with their respective type strains and repository places is provided in Table 2.

**Table 2:** Type strains of *Listeria* spp. identified since 2010

Species	Type strain (strain collection where available)	Other available strains (strain collection)
*L. marthii*	FSL S4-120^T^ (=ATCC BAA-1595^T^ = BEIR NR 9579^T^ = CCUG 56148^T^ = DSM-23813^T^)	Three other strains (BEI)
*L. rocourtiae*	CIP 109804^T^ (=DSM 22097^T^)	
*L. weihenstephanensis*	WS 4560^T^ (=DSM 24698^T^ = LMG 26374^T^)	WS 4615 (=DSM 24699 = LMG 26375)
*L. fleischmannii* subsp. *fleischmannii*	LU2006-1^T^ (=DSM 24998^T^ = LMG 26584^T^ = CIP 110547^T^)	LU2006-2; LU2006-3; DSM-25003; LMG 26585
*L. fleischmannii* subsp. *coloradonensis*	TTU M1-001^T^ (=ATCC BAA-2414^T^ = DSM 25391^T^ = CIP 110717^T^)	
*L. floridensis*	FSL S10-1187^T^ (=DSM 26687^T^ = LMG 28121^T^ = BEI NR-42632^T^)	
*L. aquatica*	FSL S10-1188^T^ (=DSM 26686^T^ = LMG 28120^T^ = BEI NR-42633^T^)	FSL S10-1181
*L. cornellensis*	TTU A1-0210^T^ (=FSL F6-0969^T^ = DSM 26689^T^ = LMG 28123^T^ = BEI NR-42630^T^)	FSL F6-0970
*L. riparia*	FSL S10-1204^T^ (=DSM 26685^T^ = LMG 28119^T^ = BEI NR-42634^T^)	FSL S10-1219
*L. grandensis*	TTU A1-0212^T^ (=FSL F6-0971^T^ = DSM 26688^T^ = LMG 28122^T^ = BEI NR-42631^T^)	
*L. newyorkensis*	FSL M6-0635^T^ (=DSM 28861^T^ = LMG 28310^T^)	FSL A5-0209
*L. booriae*	FSL A5-0281^T^ (=DSM 28860^T^ = LMG 28311^T^)	FSL A5-0279

Strain collections: *ATCC* American Type Culture Collection, *CCUG* Culture Collection University of Goteborg, *CIP* Collection of Institut Pasteur, *DSM* Deutsche Sammlung von Mikroorganismen und Zellkulturen (German Collection of Microorganisms and Cell Cultures), *LMG* Belgian Coordinated Collection of Microorganisms (BCCM/LMG), *TTU* Texas Tech University collection, *FSL* Food Safety Laboratory collection at Cornell University, *BEI* and *BEIR* BEI Resources

## Distribution

With the exception of *L. grayi*, *Listeria* sensu lato species have only recently been described and their distribution is yet to be comprehensively elucidated. However, two species, *L. newyorkensis* and *L. fleischmannii*, have already been identified in both North America and Europe (Chiara et al. 2015; den Bakker et al. 2013; Weller et al. 2015), suggesting that their occurrence may be broad, at least in the northern hemisphere. *L. newyorkensis* has been isolated from raw milk in Italy (Chiara et al. 2015) and from a seafood processing plant in northeastern USA. *L. fleischmannii* has been isolated from cheeses in Italy and Switzerland and from the environment of a cattle ranch in the state of Colorado, USA. (Bertsch et al. 2013; den Bakker et al. 2013). *Listeria* sensu lato species only isolated in Europe so far include *L. rocourtiae*, which was isolated in Austria, from processed lettuce (Leclercq et al. 2010), and *L. weihenstephanensis*, isolated from vegetation in a pond in Germany (Lang Halter et al. 2013). Species only isolated in the USA include *L. floridensis*, *L. aquatic*, and *L. riparia*, all three isolated from running waters in the state of Florida, and *L. cornellensis* and *L. grandensis*, both isolated from water in the state of Colorado (Table 3; den Bakker et al. 2014). *L. grayi* has been isolated from several continents, including Europe, Asia, Africa, South America, and North America, and sources such as freshwater fish and abattoirs (Bernagozzi et al. 1994; Bouayad et al. 2015; Hofer et al. 2000; Jallewar et al. 2007; Schlech et al. 2005), suggesting that this species is distributed globally.

**Table 3:** Reported isolation locations of *Listeria* sensu lato species (as of November 2015)

Species	Source of isolation	References
*L. rocourtiae*	Pre-cut lettuce, Salzburg (Austria)	Leclercq et al. (2010)
*L. weihenstephanensis*	Vegetation (*Lemma trisulca*) from pond in Wolnzach/Pfaffenhofen (Germany)	Lang Halter et al. (2013)
*L. fleischmannii*	Cheese and ripening cellars (Switzerland); cheese (southern Italy); environmental samples, cattle ranch, Colorado (USA)	den Bakker et al. (2013), Bertsch et al. (2013)
*L. floridensis*	Running water, Florida (USA)	den Bakker et al. (2014)
*L. aquatica*	Running water, Florida (USA)	den Bakker et al. (2014)
*L. cornellensis*	Water, Colorado (USA)	den Bakker et al. (2014)
*L. riparia*	Running water, Florida (USA)	den Bakker et al. (2014)
*L. grandensis*	Water, Colorado (USA)	den Bakker et al. (2014)
*L. newyorkensis*	Non-food-contact surface in a seafood processing plant (northeastern USA); raw milk (southern Italy)	Weller et al. (2015)
*L. booriae*	Non-food-contact surface in a dairy processing plant (northeastern USA)	Weller et al. (2015)
*L. grayi*	Various locations (worldwide)	Bernagozzi et al. (1994), Bouayad et al. (2015), Hofer et al. (2000), Jallewar et al. (2007), Schlech et al. (2005)

## Phenotypes

Like the *Listeria* sensu strictu species, all sensu lato species are catalase positive, non-spore-forming, non-capsulated, and rod-shaped. *Listeria* sensu lato species present several phenotypic characteristics that distinguish them from *Listeria* sensu strictu species (see Table 1 for details). While all sensu strictu species are Voges-Proskauer reaction positive and hence able to produce acetoin from fermentation of glucose through the butanediol pathway, among sensu lato species, only *L. grayi* and one out of two *L. aquatica* strains analyzed have this ability (den Bakker et al. 2014). Conversely, while none of the sensu strictu species are able to reduce nitrate to nitrite, all sensu lato species but *L. floridensis* can reduce nitrate (den Bakker et al. 2014; Weller et al. 2015). Motility is another characteristic that appears restricted to the *Listeria* sensu strictu species and *L. grayi*, although contradictory results were initially obtained for *L. rocourtiae* and *L. weihenstephanensis*. In initial studies, Leclercq et al. (2010) and Lang Halter et al. (2013) reported that isolates representing these species were motile, while subsequent studies (Bertsch et al. 2013; den Bakker et al. 2014) reported no motility for representatives of these species. Subsequent motility tests carried out by Weller et al. (2015) also found no motility with all sensu lato strains tested (with the exception of *L. grayi* strains) at temperatures ranging from 4 to 37 °C. These findings are consistent with genomic studies that reported absence of flagellar genes from all *Listeria* sensu lato strains (except *L. grayi*), thus suggesting that *L. grayi* is the only motile *Listeria* sensu lato species (Chiara et al. 2015; den Bakker et al. 2013; den Bakker et al. 2014).

In terms of acid production from different carbohydrate substrates, a high variability has been observed within and between sensu lato species. All sensu lato species were able to acidify D-xylose and D-glucose, but none were able to acidify glucose 1-phosphate nor inulin. Other molecules presented variable results depending on the species (Weller et al. 2015). Interestingly, the species *L. fleischmannii*, *L. aquatica*, and *L. floridensis* all seem to have lost the ability to grow in liquid media at 4 °C, a key characteristic shared among other members of the genus *Listeria*. These three species form a cluster within the genus *Listeria* and share the most common recent ancestor with *Listeria* sensu strictu species and *L. grayi*, which suggests that the inability to grow at low temperatures was lost in this group.

While molecular methods provide for most reliable species classification, some specific phenotypic characteristics can be used to differentiate each species within *Listeria* sensu lato. *L. grayi* can be distinguished from other sensu lato species by a positive result in the Voges-Proskauer test and by a positive result in a motility test (McLauchlin and Rees 2009; Weller et al. 2015). Moreover, *L. grayi* can be differentiated from *Listeria* sensu strictu species by its ability to ferment D-mannitol (McLauchlin and Rees 2009; Weller et al. 2015). *L. fleischmannii* is unable to grow well at low temperatures and can be differentiated from other sensu lato species by its ability to ferment D-arabitol, L-rhamnose, and D-ribose (Bertsch et al. 2013; den Bakker et al. 2013; Weller et al. 2015). In addition to being characterized by its inability to grow at temperatures below 7 °C, *L. floridensis* is the only sensu lato species unable to reduce nitrate (den Bakker et al. 2014; Weller et al. 2015). *L. aquatica* is the only *Listeria* species unable to ferment maltose and α-methyl D-glucoside and the only sensu lato species able to ferment D-tagatose (den Bakker et al. 2014; Weller et al. 2015). *L. newyorkensis* can be differentiated from other *Listeria* species by its inability to acidify D-arabitol, absence of α-mannosidase activity, and ability to acidify D-ribose, D-galactose, and L-arabinose (Weller et al. 2015). *L. cornellensis* can be distinguished from other sensu lato species by being unable to ferment L-rhamnose at 37 °C and presents a weak acidification of lactose (den Bakker et al. 2014; Weller et al. 2015). *L. rocourtiae* can be distinguished from other sensu lato species by its positive α-mannosidase activity combined with its inability to ferment L-arabinose and D-arabitol (Leclercq et al. 2010; Weller et al. 2015). *L. weihenstephanensis* can be differentiated from other sensu lato species by its ability to ferment D-arabitol, L-rhamnose, and D-mannitol (Lang Halter et al. 2013; Weller et al. 2015). *L. grandensis* may be differentiated from *L. cornellensis* by its stronger ability to ferment lactose (den Bakker et al. 2014; Weller et al. 2015). *L. riparia* can be distinguished from other sensu lato species by its positive α-mannosidase activity in combination with the ability to ferment L-rhamnose, D-galactose, and L-arabinose (den Bakker et al. 2014; Weller et al. 2015). *L. booriae* can be differentiated from other *Listeria* by its ability to ferment melibiose, L-arabinose, and D-arabitol (Weller et al. 2015). While a number of phenotypic characteristics can thus be used to differentiate *Listeria* sensu lato species, identification of *Listeria* sensu lato isolates using standard biochemical test kits may often not be straight forward as carbohydrate substrates that allow for species differentiation may not be included and as identification schemes and databases may not yet have been updated with information on the recently described *Listeria* species.

In terms of pathogenicity, none of the sensu lato species seem to be pathogenic as they all failed to produce positive results in both hemolytic tests and phosphoinositide phospholipase C activity tests (Bertsch et al. 2013; den Bakker et al. 2013; den Bakker et al. 2014; Lang Halter et al. 2013; Leclercq et al. 2010; Weller et al. 2015). Moreover, invasion assays using human carcinogenic cell lines (Caco-2) showed that *L. fleischmannii* is not able to internalize into human intestinal epithelial cells further supporting that this specific species is not pathogenic for humans (Bertsch et al. 2013).

## Genomic characteristics

**Fig. 2:**
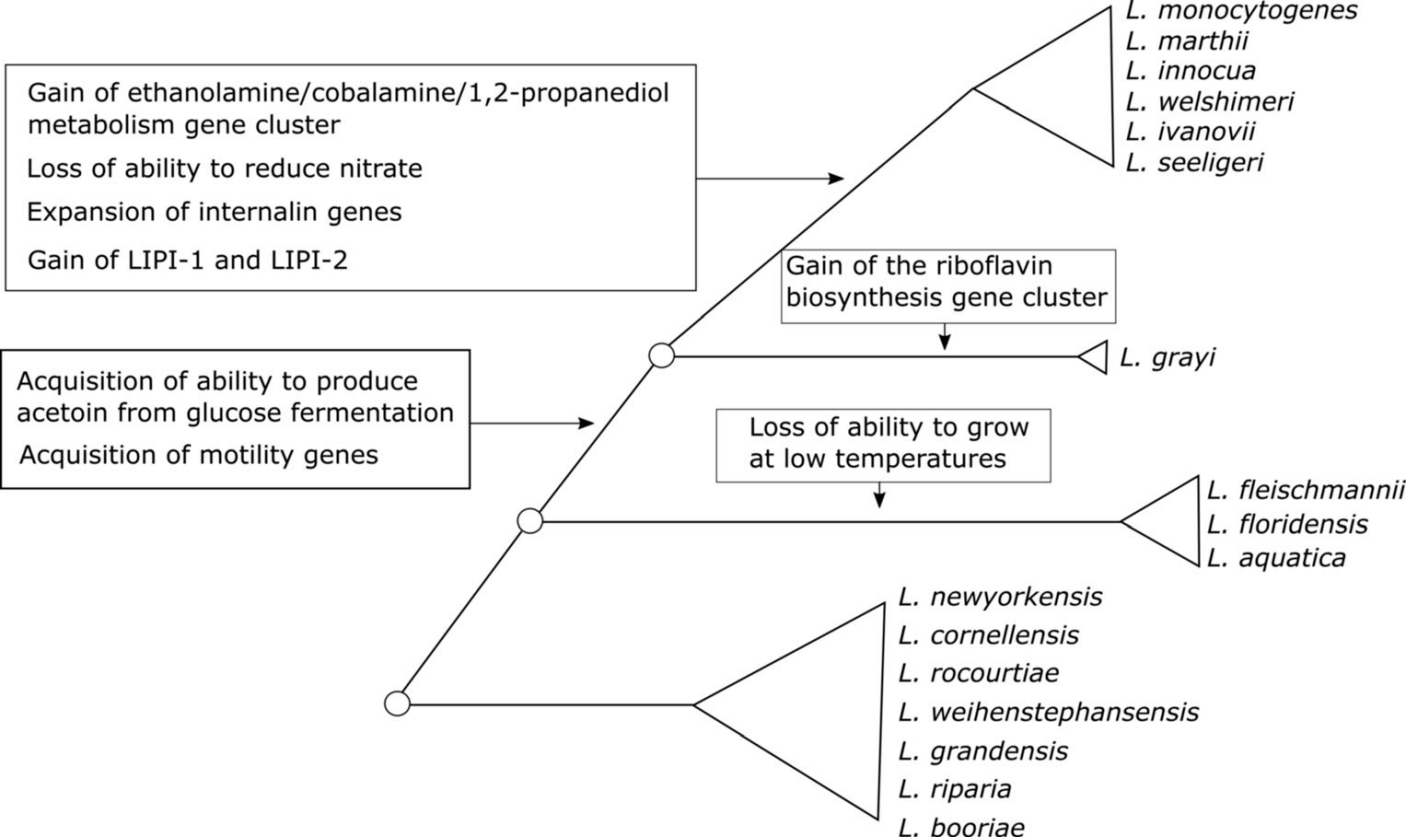
Schematic of the phylogenetic history of the current genus *Listeria*. *Circles* represent extinct ancestor species. *Triangles* represent the current monophyletic taxa proposed here to be classified into distinct genera. *Text close to branches* depicts evolutionary events of gain or loss of genetic features that help define current taxa

The availability of genome sequence data for all *Listeria* sensu lato species not only presented unique opportunities to better characterize these species but also allowed for novel insights in the evolution of virulence related and other relevant phenotypes in the overall genus *Listeria* (Fig. 2). The G+C content of sensu lato genomes varies from 38.3 % (*L. fleischmannii*) to 45.2 (*L. newyorkensis* and *L. booriae*). As expected based on phenotypic data, genome analyses showed that none of the *Listeria* sensu lato species harbor the *Listeria* pathogenicity island 1, which includes the major virulence genes *prfA*, *plcA*, *hly*, *mpl*, *actA*, *plcB*, or the *Listeria* pathogenicity island 2, which includes *inlA* and *inlB*. With the exception of *L. grayi*, none of the other sensu lato species carry the motility genes that encode flagellar proteins, which explains why these species are not motile. Phylogenetic analyses suggest that the ancestor of *Listeria* sensu strictu and *L. grayi* acquired the whole flagellar biosynthetic genes through horizontal gene transfer from an ancestor of the *Bacillus cereus* complex (Chiara et al. 2015) (see Fig. 2). The only gene related to motility that is present in a sensu lato species (other than *L. grayi*) is *mogR*, which was identified only in *L. fleischmannii*. This gene encodes for the motility gene repressor MogR (den Bakker et al. 2013); its specific function in this species remains to be determined. Another interesting genomic feature was observed among the genome sequences for two *L. grayi* strains. Four genes, genomically clustered, were found among these strains and encode for proteins involved in the biosynthesis of riboflavin (Chiara et al. 2015). The origin of these genes is not clear. However, the absence of these genes from all other *Listeria* species suggests that the cluster was acquired via horizontal gene transfer by the ancestor of *L. grayi* from another bacterial species.

Interestingly, *Listeria* sensu lato species genomes showed an underrepresentation of genes associated with internalin domains when compared to sensu strictu species (Chiara et al. 2015; den Bakker et al. 2010b). This suggests that expansion of internalin genes happened after the divergence between *Listeria* sensu strictu and *Listeria* sensu lato and further supports that *Listeria* sensu lato strains are unlikely to be pathogenic for mammalian species as internalins have been shown to typically be important for interactions between *Listeria* and mammalian hosts (Pizarro-Cerda et al. 2012). Interestingly, *L. fleischmannii* subsp. *coloradonensis* genome has also been found to harbor a gene encoding a putative mosquitocidal toxin MTX2 from *Bacillus*, which also resembles the *Clostridium perfringens* potent epsilon toxin ETX that causes rapid fatal enterotoxemia in animals (den Bakker et al. 2013); this raises the possibility that at least some sensu lato isolates may be able to cause disease in non-mammalian hosts such as insects.

## Phylogeny and phenotypic characteristics of *Listeria* sensu lato suggesting that these species may represent new genera

In taxonomy, delineation of different species is governed by fairly well-defined criteria. While, traditionally, DNA-DNA hybridization values of <70 % were used to delineate different species (Wayne et al. 1987), more recently whole genome sequencing-based criteria (ANIb) have been used to define different species (Richter and Rossello-Mora 2009). In *Listeria*, this approach was used by den Bakker et al. (2014) and Weller et al. (2015) to classify newly identified *Listeria* strains into seven distinct species and to confirm classification into separate species for other species that had been proposed previously (Bertsch et al. 2013; Lang Halter et al. 2013; Leclercq et al. 2010).

While clear cutoffs exist for classification of isolates into a new species, cutoffs for defining new genera are much less well defined (Qin et al. 2014). It has been suggested that a 16S rRNA diversity higher than 5 % or an amino acid identity (AAI) lower than 70 % could be used to determine whether two species should belong to distinct genera (Konstantinidis and Tiedje 2007). Qin et al. (2014) have suggested that a percentage of conserved proteins (POCP) > 50 % should be used as a threshold to define whether two species should be classified into the same genus or not. Deloger et al. (2009) suggested the utilization of the DNA maximal unique matches (MUMs) shared by two genomes as a tool for classification of species and genus. However, there is no clear agreement on definitive thresholds that could be used to determine boundaries of bacterial genera. A clear convergence of phylogenetic and phenotypic data as well as whole genome sequencing-based similarity measures suggested though that the current genus *Listeria* may warrant reclassification into more than one genus. *Listeria* sensu strictu clearly represents a distinct and well-defined group of organisms that should be maintained as a single genus with the name *Listeria*. The species currently grouped in *Listeria* sensu lato on the other hand appear to warrant reclassification into three different genera (see Fig. 1); reclassification into more than one genus is not only required to avoid phylogenetically incongruent genera but also yields proposed genera with distinct and unique phenotypic characteristics (e.g., all species in the proposed genus *Mesolisteria* are not able to grow at 4 °C). Specifically, we propose that *L. grayi* could be classified into a distinct new genus named *Murraya*; this represents a revival of the previously proposed genus *Murraya* (Wemekamp-Kamphuis et al. 2002). ANIb values based on comparisons of genome sequences of *Murraya* strains and *Listeria* sensu stricto strains support that these taxa are highly divergent (ANIb <73 %). *L. fleischmannii*, *L. aquatica*, and *L. floridensis* could be classified into another genus named *Mesolisteria* (referring to the mesophillic nature of species within this genus). Finally, the species *L. newyorkensis*, *L. cornellensis*, *L. rocourtiae*, *L. weihenstephanensis*, *L. grandensis*, *L. riparia*, and *L. booriae* could be classified into the new genus named *Paenilisteria* (almost *Listeria*, referring to the phenotypic resemblance to the genus *Listeria*). Importantly, *Listeria* sensu lato species present some phenotypic features different from those that characterize the genus *Listeria*. For example, with the exception of *L. grayi*, all sensu lato species are non-motile, while all sensu strictu species are motile. All sensu lato species with the exception of *L. floridensis* are capable of reducing nitrate to nitrite, an ability not present among sensu strictu species. Moreover, three sensu lato species (*L. fleischmannii*, *L. floridensis*, and *L. aquatica*) are unable to grow at low temperatures, one of the major characteristics of the genus *Listeria*. Genomically, sensu lato species also lack features characteristic of the genus *Listeria* such as a high number of genes encoding for internalin-like proteins. Based on the data available to date, we thus would like to propose that the *Listeria* sensu lato species may warrant classification into separate genera according to their phylogenetic and phenotypic characteristics (Fig. 2), even though further genome sequence analyses (e.g., determination of AAI, POCP, and MUM values) may be helpful and needed for a final reclassification proposal.

## Conclusions

The genus *Listeria* has considerably expanded in the past decade to include 17 species with diverse phenotypic and genotypic characteristics. Comparative characterization of the nine species newly described since 2009, including through comparative genomic analyses, has provided new insights into the evolution of *Listeria* and suggests a need to reevaluate the taxonomy of the current genus *Listeria*. Specifically, there is convincing evidence that a group of *Listeria* species (“*Listeria* sensu lato”) that are distinct from *L. monocytogenes* may warrant reclassification into three different genera (including revival of the previously proposed genus *Murraya*); these species include all but one of the *Listeria* species newly reported since 2009. Taxonomy revaluation of *Listeria* sensu lato and clarification of the genetic and phenotypic characteristics of this group are of particular importance, since this group includes species that show distinct phenotypes from *Listeria* sensu strictu, which includes the pathogen *L. monocytogenes*. For example, all *Listeria* sensu lato species, expect for *L. grayi*, lack flagellar motility, and a distinct subset of *Listeria* sensu lato species (proposed to represent the new genus *Mesolisteria*) lacks the ability to grow at temperatures below 7 °C. Identification of a number of new *Listeria* species that are distinct from *Listeria* sensu strictu (which includes *L. monocytogenes*) and that have preliminarily been designated as *Listeria* sensu lato also is important for diagnostic kit manufacturers and the food industry, which uses tests for the genus *Listeria* as an indicator that detects conditions that allow for presence, growth, and persistence of *L. monocytogenes*. In particular, clarity is needed whether a meaningful test for conditions that allow for presence, growth, and persistence of *L. monocytogenes* should (i) detect only *Listeria* sensu strictu or (ii) should detect all 17 currently described *Listeria* species. Reclassification of *Listeria* sensu lato into different genera would provide clarity on this issue and would clarify the genus *Listeria* as a genetically and phenotypically circumscribed group that only contains six species that share key phenotypes such as ability to grow at low temperatures and a positive Voges-Proskauer reaction. Until a reclassification and clarification of the genus *Listeria* is completed, it is essential for end users of *Listeria* tests to understand which *Listeria* species a given test detects and to correspondingly interpret test results (e.g., whether detection of *Listeria* spp. that do not grow at refrigeration temperatures truly predicts conditions that allow for presence, growth, and persistence of *L. monocytogenes*).
